# Robustness and Fragility in Immunosenescence

**DOI:** 10.1371/journal.pcbi.0020160

**Published:** 2006-11-24

**Authors:** Sean P Stromberg, Jean Carlson

**Affiliations:** Physics Department, University of California Santa Barbara, Santa Barbara, California, United States of America; Rice University, United States of America

## Abstract

We construct a model to study tradeoffs associated with aging in the adaptive immune system, focusing on cumulative effects of replacing naive cells with memory cells. Binding affinities are characterized by a stochastic shape space model. System loss arising from an individual infection is associated with disease severity, as measured by the total antigen population over the course of an infection. We monitor evolution of cell populations on the shape space over a string of infections, and find that the distribution of losses becomes increasingly heavy-tailed with time. Initially this lowers the average loss: the memory cell population becomes tuned to the history of past exposures, reducing the loss of the system when subjected to a second, similar infection. This is accompanied by a corresponding increase in vulnerability to novel infections, which ultimately causes the expected loss to increase due to overspecialization, leading to increasing fragility with age (i.e., immunosenescence). In our model, immunosenescence is not the result of a performance degradation of some specific lymphocyte, but rather a natural consequence of the built-in mechanisms for system adaptation. This “robust, yet fragile” behavior is a key signature of Highly Optimized Tolerance.

## Synopsis

The immune system can be viewed as a complex system, which adapts, over time, to reflect the history of infections experienced by the organism. This paper describes a model that captures this adaptation and corresponding robust, yet fragile behavior. To model immunological processes that rely on binding specificity, researchers typically utilize abstract shape space models. These models describe the binding characteristics of a receptor or antigen as points in a high dimensional vector space. Stromberg and Carlson have incorporated the concept of shape space into a dynamical model of immune response. They use this model to examine the development of the system over a series of infections and monitor the severity of disease for each infection. The diseases are drawn at random from a distribution having a few frequently reoccurring and many rare. The system is observed to adapt over a series of infections, becoming robust to the frequent diseases while developing fragility to the rare diseases. This age-correlated weakness arises from the underlying dynamics of system adaptation rather than from an accumulation of defects. This robust, yet fragile behavior is a signature of Highly Optimized Tolerance, a mechanism for complexity based on robustness tradeoffs.

## Introduction

The adaptive immune system [1] of vertebrates has evolved in a manner that enables adaptation to the history of infections over the lifetime of each individual organism. It consists of a complex, heterogeneous collection of cells that is derived from stem cells in the bone marrow and proliferates in the lymph nodes. These cells are endowed with the remarkable ability to discriminate between self and nonself agents within the body and to remove the nonself elements [2–4]. B and T cells are the white blood cells (i.e., lymphocytes) that constitute the adaptive components of the immune system. They derive their ability to discriminate self from nonself with the binding specificity of their receptors: T cell receptors for T cells, and membrane-bound antibody for B cells. These receptors are assembled randomly from gene segments, producing a population of naive cells, in which each individual combination has a different binding specificity. The random combinations of genes give the immune system the ability to produce diverse cells capable of responding to many pathogens. During an infection, the cells whose receptors recognize the antigen proliferate and differentiate into antigen-removing effector cells and long-lived memory cells. The memory cells give rise to a more rapid and efficient response to a secondary exposure to the same antigen. However, due to homeostatic regulation of the lymphocyte population, the growth of memory cells reduces the naive cell population size. Over time, this has the effect of increasing sensitivity to novel infections.

We introduce a model that captures this tradeoff between resilience to repeated exposure and sensitivity to new pathogens. The model consists of coupled differential equations for immune-system cell populations, defined in terms of their primary immunological function and their binding characteristics. The relative population sizes evolve in time, stimulated by episodic infections. Antigens are drawn from a probability distribution of their characteristics, which enables estimation of the binding affinity of lymphocytes. We include a constraint on the total number of immune cells in the system, and define an immunological loss function that quantifies disease severity. The constraint on the number of cells implies that memory, which is specific to infections the system has seen, comes at a price for unseen infections [5]. Our model illustrates how the immune system initially increases in effectiveness but eventually becomes overspecialized with age.

## Results

Characteristics of the distinct populations in our model are summarized in Table 1. Note that in our simplified model, as in Segel and Pereleson [4], lymphocytes (memory, naïve, and effector cells) are not specifically T or B cells, but a generalization having properties common to both types. We have also omitted helper T cells (that help to stimulate the immune response), as well as the complex germinal center reaction and somatic hypermutation (processes involving the proliferation and development of lymphocytes), assuming these features are not limiting factors in immunosenescence.

**Table 1:**
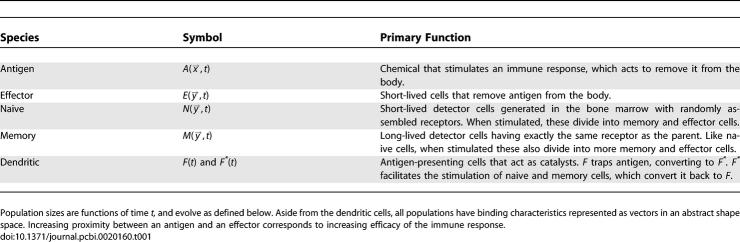
Immune System Model Ingredients

In our model, *A, E, N,* and *M* are all fields on a generalized shape space, introduced by Oster and Perelson [6] to represent the lock-and-key type specificity of antigen-receptor binding. The dimensions of the generalized Euclidean shape space correspond to quantities such as size, charge distribution, and hydrophobicity. This differs from other Hamming-type shape space models where each dimension pertains to a particular amino acid in the binding-region sequence [7]. The binding sites on the antigens and receptor proteins are described by the position vectors in the shape space, x⃗ and y⃗, respectively. The binding of antigen to dendritic cells as described in Table 1, is not shape space–dependent. All antigens in this model bind to dendritic cells with the same affinity. Recent calculations indicate that the shape space is best described with between five and eight dimensions [7]. We use two dimensions here for visualization. Using higher dimensions in the model changes the distribution of affinities, but does not dramatically affect the results of the paper. Extensions to higher dimensions, as well as to the more complex interactions listed above, will be considered in future work.

Vector values of the antigen x⃗ and immune cells y⃗ describe complementary binding characteristics, so that the binding affinity for *A*(x⃗) and a receptor at y⃗, given by γ(x⃗, y⃗), is maximal for x⃗ = y⃗. For x⃗ ≠ y⃗, the binding affinity is a decaying function of the distance from x⃗ to y⃗ in the shape space γ = γ(|x⃗ − y⃗|). Following Segel and Pereleson [4], we take the affinity function to be a Gaussian:

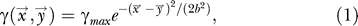
where *γ_max_* sets the overall scale for the strength of the immune response, and *b* sets the mismatch tolerance between antigens and receptors. Replacing *γ* with different decaying functions of distance (e.g., exponential) does not significantly alter the results of this work.

Using the species listed in Table 1, we next define our model of immune system response and adaptation. The process can be broken down into three stages: (i) antigen proliferation and immune response in an individual infection, (ii) recovery and stasis between infections, (iii) long-term adaptation of lymphocyte populations over the lifetime of the individual. We assume that rates of infection are small enough that the immune system completely eliminates one pathogen, and relaxes to the uninfected state long before the next infection occurs. This allows us to introduce our model in three stages, corresponding to the increasing time scales (i)–(iii) above, beginning with an individual infection.

Periods of infection are associated with introduction of antigen. Different diseases are associated with different shape space coordinates x⃗, and have different rates of infection. Upon infection, a pathogen proliferates at an exponential rate so that the antigen population grows at a rate β*A*(x⃗) (Equation 2) In our model, *t* = 0 marks the time when the pathogen is mixed into the lymph and begins to stimulate an immune response. We assume a finite value of *A*(x⃗,0) at this onset to account for the delay in the start of the immune response. This represents how once a small amount of the pathogen bypasses the physical barriers of the innate immune system it will proliferate until it finds its way into the blood and then lymph nodes, at which point the immune response is triggered.

Next the unoccupied dendritic cells, *F,* begin to trap antigen and become activated to F^*^ at a rate ρ*FA*(x⃗) (Equations 2 and 3). The activated dendritic cells F^*^ now present antigen to the naive and memory cells, stimulating them to divide (Equations 3–5). Overall, the stimulation occurs at a rate αγ(x⃗, y⃗)*F* ^*^(*N + M*)( y⃗). Here the factor of γ(x⃗, y⃗) gives the highest-affinity lymphocytes the most rapid stimulation. The daughters of the cellular division of either *N*( y⃗) or *M*( y⃗), are *E*( y⃗) cells with fraction *f,* or *M*( y⃗) cells with fraction (1− *f*) (Equations 4–6). Several generations of memory cells may therefore be produced through an immune response. In stimulating naive and memory cells, F^*^ reverts back to *F* (Equation 3), keeping the total *F* + F^*^ = *H* constant. This rate is the integral of the rate of *N* and *M* stimulation over the entire shape space. Effector cells eliminate antigen from the system with rate *A*(x⃗)γ(x⃗, y⃗)*E*( y⃗). The total rate of antigen removal is the integral of this rate over the shape space of effector cells (Equation 2). Effector cells are short-lived and die with rate δ*E*( y⃗) (Equation 6).

These short time scale reactions are described by the following system of equations (we drop the explicit *t* dependence in all populations to simplify notation):

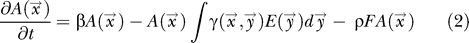


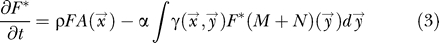


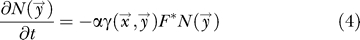


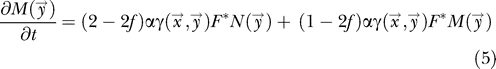


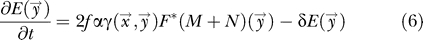



Here *α* is an affinity-independent factor that accounts for the difference in γ(x⃗, y⃗)-dependent rates of lymphocyte stimulation and removal of antigen. Note that our model does not include any spatial variables for position of antigen and lymphocytes in the body, which corresponds to assuming a well-mixed system. This system of equations exhibits many features we expect from an immune system model, such as rapid secondary response and affinity selection.

**Figure 1:**
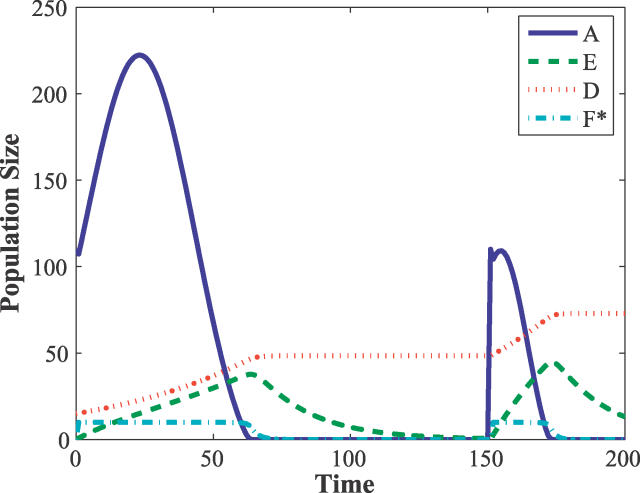
Immune Responses to Two Sequential Inoculations by the Same Antigen Results are shown for maximum binding affinity pairs x⃗ = y⃗. The rapid response to the secondary inoculation (represented by the smaller size of the second peak) is due to the elevated number of memory cells. The immunological loss, Equation 15, is defined to be the area under the antigen population peak. For the primary and secondary peaks, the values are 8,860 and 1,525, respectively. The model parameters used in this and all simulations in this paper are as follows: *α* = 1.5, *β* = 0.083, *f* = 0.38, *δ* = 0.01, *ρ* = 1, *ϕ* = 10^−4^, *H* = 10, *γ_max_* = 0.005, *b* = 2. These parameter values give typical behavior for the model.

During the immune response, the naive and memory cells are indistinguishable. In our model their difference becomes apparent on intermediate time scales. Therefore, we consider their combined effect using a single variable *D*( y⃗) = *N*( y⃗) + *M*( y⃗). Figure 1 shows a typical response to a repeated inoculation with antigen x⃗. Although other lymphocytes also bind less effectively to the antigen, for illustrative purposes we plot only populations *E*( y⃗) and *D*( y⃗) for x⃗ = y⃗, as well as F^*^ (for which binding is independent of shape space characteristics).

Initially there are 15 memory cells with x⃗ = y⃗, *D*( y⃗,0) = 15δ(x⃗ − y⃗), *F* = *H*, *E* = 0, and an antigen inoculation *A*(x⃗,0) = 110. After the first immune response is complete, there is a second identical inoculation at a later time. In each exposure, the population size of the antigen increases, until a sufficient number of effector cells are created from the memory and naive cell populations to eliminate the infection. The total number of lymphocytes *N* + *M* + *E,* increases during an immune response, corresponding to swelling of the lymph nodes. As the effectors die and the memory and naive cells are no longer stimulated, the swelling subsides. Additionally, this model predicts that symptoms associated with elevated *E* levels peak just as the pathogen is cleared. The more rapid secondary response is due to the elevated number of memory cells. (The initial steep decline in the secondary response is due to the trapping of antigen by the dendritic cells.) All other model parameters remain the same from the first exposure to the second. Between infections all short-lived effector cells die, and the F^*^ cells all revert to *F*.

To quantify the severity of an individual infection, we define a loss function *L*(x⃗) as the integral of the antigen population size with respect to time:

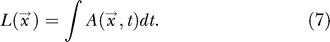



While physiologically, severity of disease depends on many factors, we believe that this is a simple natural choice, as it is a rough measure of the amount of the body's resources a pathogen may consume and the amount of toxin the pathogen may secrete. This immunological loss function serves as a tool for quantifying statistics of infection size, and provides a meaningful target for sensitivity analysis. In the context of this investigation and immunosenescence, it allows us to quantify fitness and to monitor how it changes over the development of the immune system. Additionally, loss can be used to compare the effects of additional immune system components and reactions in more detailed immune system models, and to quantify the efficacy of drugs and therapies based on their effect on loss.

We can obtain analytic estimates for loss as well as memory-cell population growth as functions of pre-infection memory and naive cell population sizes based on several simplifying approximations to Equations 2–6. For ρA ≫ αγ, F^*^ is approximately equal to the total number of dendritic cells H, F^*^ ≈ *H,* and *F* ≈ 0. Since *A* levels will be high when an immune response is initiated, this approximation is reasonable. Equations 2–6 with *M* and *N* replaced with *D* = *M* + *N,* and the approximation F^*^ ≈ *H,* reduce to:

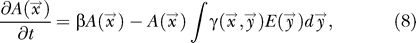


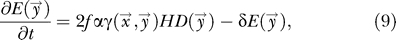


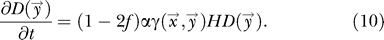



These equations can be easily integrated, yielding solutions that approximate the antigen population size during an infection. The complete expression for *A*(x⃗,*t*) is tractable, but cumbersome, and takes the form

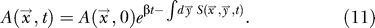



A simple expansion of the function *S*(x⃗, y⃗,*t*) to second order in *t* yields a Gaussian approximation for the *A*(x⃗,*t*) peaks (e.g., in Figure 1):

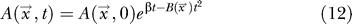
where,

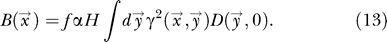



This approximation describes the *A*(x⃗,*t*) pulse as a function of the initial value of *D*.

Using this approximate solution for *A*(x⃗,*t*), we estimate the increase in memory cell–population values after the infection is cleared. We take the value of *M*( y⃗,*t*) to be constant after time *t_e_* when *A* has been reduced to half its initial value, in the tail of the pulse, and we round it to integer value.

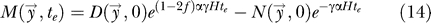



This analytical result gives close agreement with memory cell–growth levels given by our original model.

We estimate loss by integrating our analytical solution for the antigen population peak from −∞ to ∞ (rather than starting at *t* = 0) to obtain:

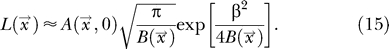



Note that extending the range of integration to −∞ makes a relatively small difference in the result and simplifies this expression. Furthermore, it may in a certain sense be more accurate, as it accounts for the proliferation of antigen before the antigen enters the lymph nodes.

On intermediate time scales the system relaxes, homeostasis adjusts naive cell number, and the naive cell population is recycled. These processes are considered fast enough to reach a steady state during the time between infections, but not so fast as to be a factor during an immune response. In the absence of antigen, the populations of effector and activated dendritic cells (which are both responsible for removing antigen from the body) relax back to zero (*E*( y⃗) = 0, F^*^ = 0, and *F* = *H*), as illustrated in Figure 1. Though during an immune response *N* and *M* cells play an identical role (represented as *D* in Figure 1), during the homeostatic period their differences become important. The memory cells are long-lived, and in the absence of antigen their population is static. Naive cells have a shorter lifetime than memory cells and die by apoptosis. As the naive cells die, homeostatic mechanisms stimulate the cells of the bone marrow to randomly repopulate the system with new naive cells. The repopulation is constrained by the total number of *D* cells, *R*:

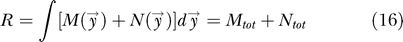



This constraint is violated during an immune response as the lymphocytes rapidly proliferate. Once the antigen is cleared, the total relaxes back to *R*. Thus, as memory cell populations rise, homeostasis effectively depletes the naive cell population. The replacement of naive cell populations with memory cells with increasing age is described by Linton and Dorshkind [8].

Next, using Equations 14–16 and simulated naive cell recycling, we study the long-term adaptation of lymphocyte populations over the lifetime of the individual. Using these approximations, our model reduces to a cellular automaton describing the population changes of lymphocytes on the shape space after each infection under our homeostatic constraint, Equation 16. Initially the system is composed of *R* naive cells. The naive cells randomly populate the shape space with uniform probability. The system is then inoculated with antigen at position x⃗ with probability *P*(x⃗). The corresponding loss is computed in Equation 15, as well as the change in the memory cell population in Equation 14. The naive cells are then redistributed with their number adjusted to satisfy Equation 16. A subsequent inoculation of the same antigen will make use of these memory cells for a more rapid response, but an inoculation at another point in shape space will have a reduced number of naive cells with which to respond and the loss will be higher.

**Figure 2:**
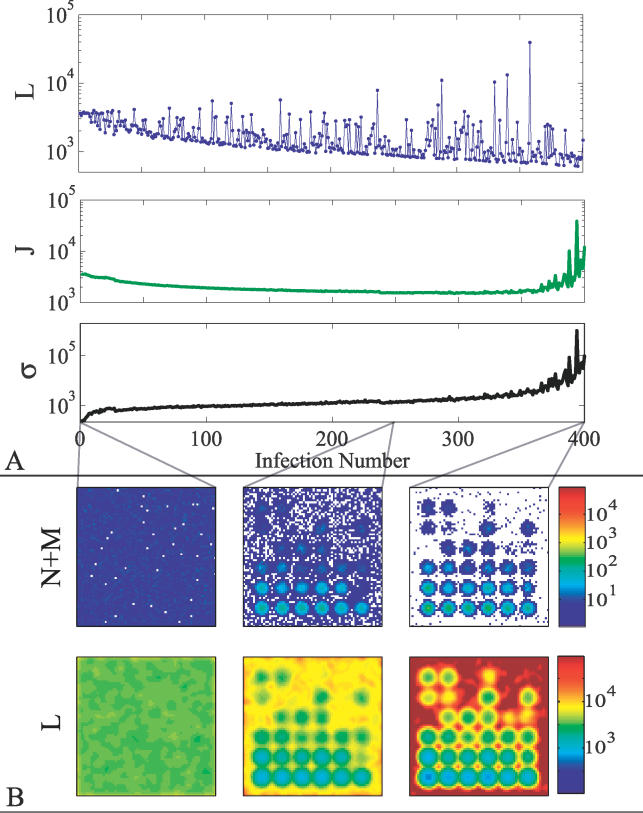
One Realization of System Development for 400 Infections on a 70 × 70 Lattice There are 36 possible infections evenly distributed throughout shape space with *R* = 33,000. The distribution of probabilities for the infections approximates an exponential, Equation 17. (A) The top curve illustrates the actual losses based on the history of infections. For most of the simulation, expected loss *J,* Equation 18, tends to decrease, yet rare events are increasing in size resulting in increased variance *σ,* Equation 19. Eventually this results in catastrophic failure. (B) Illustrates the shape space representation of populated receptor sites in the immune system initially, after 250 infections, and after 400 infections (top), and the corresponding distributions of losses for subsequent infections (bottom). The initial configuration (left) is randomly populated by naive cells. After each infection there are elevated populations of memory cells in the vicinities of the infection site. The bottom figures illustrate how the immune system becomes skewed in favor of rapid response to repeated exposures at the expense of novel infections, by illustrating the immunological losses, Equation 15, that would be incurred by inoculations at each lattice point before and after building up memory-cell populations.

We monitor the evolution of loss on long-time scales by considering a 70 × 70 lattice with *n* = 36 possible infections, at sites evenly distributed, indexed *i,* occurring with probability *p_i_.* The infections are far enough apart that cross-reactivity is not a factor. The distribution of infection probabilities is taken to have a few chronic infections that are very likely to recur, and many rare infections. The probabilities are given by an exponential distribution:

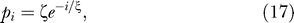
where we set *ξ* = 20/3, and 
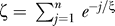
 normalizes the distribution over the discrete set of *n* infections. We choose a distribution of this form to have a mix of frequent and rare infections. We have used other distributions as well (e.g., a power law distribution), and obtained similar results. Different distributions alter the memory cell population–growth rate, which affects the time scale for the onset of immunosenescence. Realistically, the distribution of diseases and their respective infection probabilities is itself a dynamic coevolving system with new diseases constantly arising. In such a dynamic disease distribution, when the naive cell population is depleted there will be fragility similar to the observations reported here. With the kind of static distribution we consider here, in order for fragility to develop, the rare diseases must have low enough probability that one of them is likely to be experienced for the first time once the naive cell population is depleted. Changing the numerical values of parameters in the model will in general change the rate at which the naive cell population is depleted. We ran the simulation for 400 infections drawn at random from the above distribution. Figure 2A (top) shows the loss *L*(x⃗) for each event in a representative sequence.

Figure 2B (top) illustrates the corresponding lymphocyte populations (*D*( y⃗) = *M*( y⃗) + *N*( y⃗)) on the shape space at three stages in the adaptive development: initial, after 250 infections, and after 400 infections. The corresponding loss fields are illustrated below (these illustrate the loss that would be incurred for a subsequent infection as a function of the antigen characteristics x⃗). In Figure 2B, top left, *D* is strictly composed of naive cells. In Figure 2B, top middle, *D* includes a mix of memory cells that form in the neighborhood of the inoculations and the recycled naive cells. The rightmost figure is almost entirely depleted of naive cells. The bottom images show *L*(x⃗) for the naive state and after inoculations. Figure 2B, bottom, illustrates what the loss would be, given the *D* values in Figure 2B, top, for an inoculation at each point on the lattice (though we only consider the 36 points to be possible infections). Initially there are few vulnerabilities, associated with potentially large losses (red), in the system. Instead, the system is uniformly protected. However, after 250 infections, the system develops structure and has areas of high potential loss around the rare antigens. The points around the most common infections are well-protected after 250 infections, indicating low values of loss (dark blue) in subsequent infections. However, because of the overall constraint on the number of cells, many outlying areas are left more vulnerable than they were initially.

Based on the probability distribution of infections, we calculate the expected loss *J* at each stage of the system's adaptation. Here *J* corresponds to the average value of *L*(x⃗) computed over the full spectrum of possible infections and weighted according to the probability of each infection:

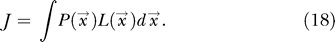



The standard deviation *σ* gives a measure of the corresponding variation in the possible loss values:

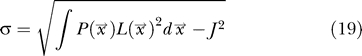



The expected loss *J* (Figure 2A, middle) initially decreases from the starting value, associated with the random population of shape space. As the system adapts, *J* takes its minimum value at roughly 250 infections, which we refer to as the “optimal” state. In later stages *J* begins to rise, due to overspecialization. It is this increase that we associate with immunosenescence. Throughout the simulation, adaptation is accompanied by a steady increase in the variability *σ* (Figure 2A, bottom), associated with increasing breadth in the distribution of losses as the system becomes increasingly specialized. At the latest stages of the simulation the increase in *σ* sharpens, which is indicative of extreme vulnerability to rare events.

**Figure 3:**
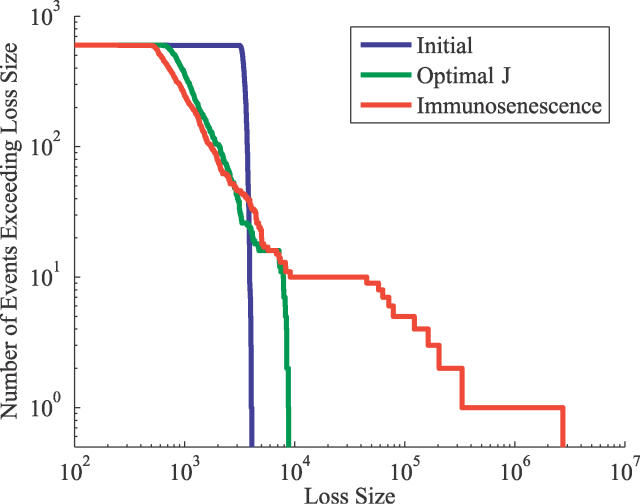
Distribution of Losses over 600 Realizations of the System Results are shown after the first infection, after 250 infections (when J is at its lowest value, corresponding to the optimal state), and after 400 infections (corresponding to immunsenescence), in blue, green, and red respectively. The infection probabilities have the same distribution as in Figure 2.

Figure 3 illustrates the cumulative statistical distribution of loss sizes obtained by combining data from 600 simulations of the form illustrated in Figure 2. The initial state is characterized by a narrow (note the logarithmic axes) and flat distribution, which reflects the uniform coverage of shape space by the random population of naive cells. The green curve corresponds to the optimal state, where the expected loss *J* takes its minimum value. Compared with the initial state, here the distribution of losses is both broader and more variable, indicative of adaptation that optimizes the inherent tradeoff between reducing loss sizes for frequent events, at the cost of larger losses for less frequent infections, which arises because of the overall resource constraint (Equation 16). The red curve shows the result at the end of our simulation, when the system has overspecialized, and exhibits immunosenescence. In this case, the distribution of losses is extremely heavy-tailed, corresponding to the increase in *J*. Any distribution containing very rare events leads to heavy-tailed loss statistics as the naive cell population becomes depleted. This heavy-tailed distribution of loss shows immunosenescence in the increased fragility of an aged immune system to as yet unseen diseases.

## Discussion

Our model is representative of the HOT mechanism [9,10], in which robustness tradeoffs provide a mechanism for complexity and power laws through either deliberate design or biological evolution, both of which favor configurations that minimize loss (Equation 18) subject to resource constraints (Equation 16). The simplest examples are referred to as “Probability Loss Resource” (PLR) HOT models [11–13], which incorporate physically motivated relationships between resource allocations and loss sizes of individual events to define a constrained resource optimization problem involving a set of events with prescribed probabilities. In the cases that have been studied to date, resources have acted as barriers to propagation of cascading events, such as wildfires [14] or power outages [15]. In our case, the analogy is more akin to a sprinkler system, populated by lymphocytes, in the shape space of possible pathogens. Over time, adaptation leads to specialized states, through replacement of naive cells with memory cells, which are tuned to the history of past exposures. This results in a system that is increasingly robust to common disturbances, yet increasingly fragile to rare events—a key signature of HOT. In our model, this age-correlated effect is a result of overspecialization rather than of an accumulation of defects. Other possible factors, such as deterioration, may contribute to immunosenescence as well, though it has been experimentally observed that some symptoms are due to system dynamics [16].

Consequences of overspecialization were studied previously in a HOT model of evolution, based on Darwinian mechanisms [17,18], leading to extreme vulnerability, similar to our observations here. In that case, offspring of lattice organisms evolved through random mutation relative to their parent lattice, and fitness was based on disturbances over the lifetime of individual lattices. Competition resulted in development of generalists and specialists. While specialists flourished during common circumstances, they experienced episodic extinction during rare events, which parallels the extreme fragility in our model associated with immunosenescence. In that case, the mutation rate itself was subject to mutation, and high mutation rates played an important role in rapid diversification and evolution following an extinction of the specialists. In the immune system, rapid mutation is associated with somatic hypermutation, which gives the daughter cells of lymphocyte stimulation a receptor that is a mutation of the parent's corresponding receptor. This gives rise to higher affinity and more efficient responses [19], and will be considered in future work.

While we have focused on immunosenescence, there are numerous additional robustness tradeoffs associated with the immune system. For example, the immune system has the ability to attack and remove nonself elements from the body with no prior knowledge of nonself features. Normally this is done with little harm to the body itself. However, the immune system can make mistakes in recognition, leading to autoimmune disease, a fragility which would not be present if an organism had no immune system to begin with. In addition, on adaptive time scales, the ability to retain memory of past exposures enables development of effective vaccines, and reduces the severity of outbreaks of communicable diseases within populations. However, in some instances vaccinations may also lead to increased susceptibility to similar diseases [19,20]. This “robust yet fragile” behavior is a key feature of HOT, a statistical theory for complexity in designed, evolved, or adaptive systems. The immune system can be viewed as a complex system in which robustness tradeoffs play a central role in evolution of the basic operating mechanisms as well as adaptation of cell populations within an individual. We emphasize the importance of tradeoffs associated with a spectrum of possible events. Evolution and adaptation favor increased robustness to common disturbances, but this is inevitably paired with increased fragility, both to rare events and to new opportunities for diseases and disturbances to hijack the system, which would not be available were the system not in place.

## Materials and Methods

All computations and stochastic and numerical simulations were done using MATLAB (The Mathworks, http://www.mathworks.com) on a personal computer.

## Abbreviations

HOT: Highly Optimized Tolerance
